# Diagnostic and Therapeutic Uses of the Microbiome in the Field of Oncology

**DOI:** 10.7759/cureus.24890

**Published:** 2022-05-10

**Authors:** Manasa Anipindi, Daniel Bitetto

**Affiliations:** 1 Internal Medicine, Einstein Medical Center Philadelphia, East Norriton, USA

**Keywords:** treatment, diagnosis, cancer, infection, microbiome

## Abstract

Cancer is a leading cause of death worldwide and it can affect almost every part of the human body. Effective screening and early diagnosis of cancers is extremely difficult due to the multifactorial etiology of the disease and delayed presentation of the patients. The available treatments are usually not specific to the affected organ system, leading to intolerable systemic side effects and early withdrawal from therapies. In vivo and in vitro studies have revealed an association of specific microbiome signatures with individual cancers. The cancer-related human microbiome has also been shown to affect the response of tissues to chemotherapy, immunotherapy, and radiation. This is an excellent opportunity for us to design specific screening markers using the microbiome to prevent cancers and diagnose them early. We can also develop precise treatments that can target cancer-affected specific organ systems and probably use a lesser dose of chemotherapy or radiation for the same effect. This prevents adverse effects and early cessation of treatments. However, we need further studies to exactly clarify and characterize these associations. In this review article, we focus on the association of the microbiome with individual cancers and highlight its future role in cancer screenings, diagnosis, prognosis, and treatments.

## Introduction and background

Cancers take a major toll on life globally. There were 10 million deaths from cancer worldwide in 2020 [1]. There has always been a search for the cause and new treatments. The causes of cancers are multifactorial processes involving genetic and environmental factors, most of which are unmanageable. There have been numerous reports of the relationship between microbial dysbiosis and cancer through chronic inflammation. This association between inflammation and cancer was first described as early as 1863 by Virchow [2]. Rous and Kidd postulated for the first time that cancers originate from subthreshold neoplastic states caused by somatic mutations induced by viral and chemical carcinogens [3]. The DNA alterations in the initiation state can be persistent, leading to a second stimulation called the promotion state. The link between microbial chronic inflammation and malignancy is well studied in colorectal cancer associated with inflammatory bowel diseases [4].

The human body has about 40 trillion cells and 22,000 human genes. We also are a host of 100 trillion microbial cells together called microbiota. There are about two million microbial genes in the microbiota, together called the metagenome [5]. The collection of genomes of all the microorganisms is named the microbiome. Microbial gene count surpasses the human genome gene count by approximately 100 times [6]. Microbiomes in human hosts live in close relationships and benefit the host in many ways. Commensal microbiota in the human body is particularly important in several functions. For example, in the gastrointestinal system, the microbiome helps in the absorption of nutrients. They protect against pathologic bacteria and play a key role in the regulation of the host immune system [7]. This intricate relationship can sometimes lead to disease development, especially when there are changes in homeostasis. Microbiome changes are linked to allergic diseases [8], diabetes [9], obesity [10], atherosclerosis [11], inflammatory bowel diseases [12], inflammatory disorders [13], and cancers. The variations in the microbiome are due to several external environmental and internal factors. For example, the salivary microbiome is usually affected by oral health, denture use, smoking, coffee, and tea consumption [14].

Direct link of infection with cancer

In 2018, 2.2 million new cancer cases were due to infections, accounting for about 13% of the global cancer burden [15]. Many viruses directly cause cancers. Examples include lymphomas caused by Epstein-Barr virus; liver cancer caused by hepatitis B virus; adult T cell leukemia caused by human T lymphotropic virus-1; cervical, vulvar, vaginal, penile, and anal cancers caused by human papillomavirus (HPV); hepatitis C virus causing liver cancer, non-Hodgkin’s Lymphoma, cancer of bile ducts, pancreatic, and head and neck cancers [16]. The bacterial species directly associated with cancers include Salmonella typhi (S. typhi) with gall bladder cancer [17,18], Streptococcus bovis with colon cancer [19,20], Chlamydia pneumoniae with lung cancer [21,22], and Helicobacter pylori (H. pylori) with gastric cancer [23,24]. There is increasing evidence to show that cancers are not only caused by a single pathogen but are also related to changes in our microbiome. Infection with bacteria or viruses leads to chronic inflammation, microbial dysbiosis, and cancers. Microbial dysbiosis has been associated with various cancers including esophageal, gastric, colon, pancreatic, breast, gall bladder, and others. Even though microbes are commonly seen as an etiology of cancer, several microbes have immune-stimulatory properties, especially gut microbiota [6].

The human microbiome

The human microbiota includes bacteria, viruses, fungi, and protozoa. Of the entire microbial mass in the human body, about 99% is in the gastrointestinal tract and it has both local and distant effects. Even though there are specific small microbiomes, the gastrointestinal microbiome is the most investigated one in our entire body given its overall size [25]. It has the highest effect on our overall health as the gut microbiome is separated from all major organs by a very thin epithelial layer. Hence, the host immune system is especially important in maintaining homeostasis in the gut, and dysbiosis can lead to chronic inflammation throughout the body [26]. Dysbiosis in the oral and gut microbiome is associated with carcinogenesis in many organs. There are differences in the density of the microbiome within each organ, which might be indirectly related to the rate of cancer in that area. For example, compared to the small intestine, the large intestine has a higher density of microbiome, which indirectly correlates with the high rates of cancer seen in the large intestine [27]. Even though organs like the liver do not have any specific microbiome, these cells are exposed to the gut microbiome, specifically microorganism-associated molecular patterns (MAMPs) and other bacterial metabolites [28,29].

The human microbiome plays a vital role in human health and diseases by its unique effect on immunity, inflammation, and metabolism [30]. Animal studies have shown that killing bacteria in the gut using antibiotics also leads to the development of cancers in the liver and colon. Similarly, there has been evidence showing the relationship between microbiota and the development of cancers in organs like skin, breasts, and lungs. The microbiome regulates primary and secondary lymphoid tissue activities through antigenic mimicry, cytokine signaling, and other metabolic effects, showing powerful antitumor effects by acting as toll-like receptor and NOD-like receptor agonists [31,32]. Microbial metabolites have been shown to affect tumor somatic mutations and immune checkpoint inhibitor efficacy [6]. Several ongoing preclinical trials are evaluating the role of microbiota in cancer treatment [6].

## Review

The association of the human microbiome with individual cancers

Head and Neck Cancers

Head and neck squamous cell cancers are the cancers of the mucosal lining of the oral cavity, pharynx, and larynx. It was responsible for 450,000 deaths worldwide in 2018 [33]. Even though tobacco consumption, alcohol abuse, and infection with HPV remain the major risk factors, there has been increasing evidence to suggest that people who are not exposed to these risk factors are also affected. Our oral microbiome has 750 distinct species of organisms that not only include bacteria but also viruses, fungi, archaea, and protozoa. Dysbiosis in this microbiome leads to a chronic inflammatory state, suppresses the anti-tumor immunity, and leads to the creation of novel mutagens. One of the examples supporting this evidence is periodontitis, which is associated with an increased risk for cancer and poor survival in many studies [34].

Oral Cancers

American Cancer Society estimates that there were about 54,000 new cases of oral cancer, leading to 11,230 deaths, in the United States in the year 2022 [35]. Oral squamous cell carcinoma is the most common oral cancer, accounting for 2% of all cancers and with a higher recurrence rate even with treatment [36]. In animal studies, Porphyromonas gingivalis and Fusobacterium nucleatum were associated with the development of oral cancers [37]. Infection with HPV increases the risk of oral squamous cell cancers and esophageal cancer [38,39]. Periodontitis is associated with oropharyngeal and esophageal cancers [40,41]. The bacteria that are shown to be increased in oral squamous cell carcinoma include Streptococcus, Fusobacterium, Capnocytophaga, Prevotella, and others [42-45]. Changes in microbiota were noted in throat cancer patients as well [46].

Hematological Cancers

Microbiome changes in the oral cavity and gut play a key role in the prognosis and treatment of cancers including lymphomas, leukemias, and multiple myeloma [47]. The small molecules produced by the gut microbiota play a vital role in modulating the immune system. Patients with periodontal disease have a higher incidence of hematopoietic and lymphatic cancers [48]. Pretreatment gut microbiome is much different in composition and less diverse in diffuse large B cell lymphoma patients compared to controls. This microbiome also played a role in predicting treatment outcomes [49]. H. pylori-associated gastric mucosa-associated lymphoid tissue lymphomas (MALT lymphomas) are usually treated with just antibiotics leading to clinical remission [50,51]. Changes in the composition of microbiota are associated with T-cell lymphoma, cutaneous B-cell lymphoma, MALT, and Hodgkin’s lymphomas [52,53]. Gut microbiota also influences chemotherapy toxicity. Bacteria can affect the response to anticancer drugs positively or negatively. Reports are showing increased cytotoxicity to one group of chemotherapeutic agents and decreased side effects of others [54]. The diversity of the microbiome influences prognosis after allogeneic stem cell transplantation, which is a potentially curative strategy for high-risk hematological malignancies [55,56]. The gut microbiome influences the treatment of multiple myeloma [57] and acute myeloid leukemia [58]. Differences in the composition of gut microbiota have been shown to lead to changes in the rates of development of graft versus host disease (GVHD) [59,60]. Increased abundance of gut commensal Blautia has been associated with reduced GVHD-induced mortality [61].

Cancers of the Respiratory Tract

Laryngeal Cancers

Laryngeal cancer accounts for about 1% of all new cancer cases and deaths globally [62]. The throat microbiome composition was different in patients with laryngeal cancer compared to controls [63]. In laryngeal cancer, tumor tissues showed higher alpha diversity and microbiota profiles compared to controls [64].

Lung Cancers

Lung cancer is the leading cause of cancer death with an estimated 1.8 million deaths worldwide [1]. The five-year survival rate is less than 20%. Even though lung tissue was once considered a sterile environment, with recent next-generation sequencing technology, it has been found that microbes play a vital role in maintaining the ecological balance in the lung [65]. The lung microbiota is a combination of bacteria, viruses, and fungi inhaled from the upper airways [65]. Bacteroides, Firmicutes, and Proteobacteria along with Streptococcus, Pseudomonas, Veillonella, and Prevotella are the most common organisms found in the lung. Destruction of the host mucosal balance can lead to microbial dysbiosis, which in turn leads to increased susceptibility to cancer through chronic inflammation [66,67]. Studies also demonstrated changes in the microbiome of gut and saliva in lung cancer patients compared to controls [68,69]. Preclinical studies show that germ-free mice and antibiotic-treated mice are less susceptible to lung cancer development induced by KRAS mutation and p53 loss [70].

Thyroid Cancer

Thyroid cancer is the ninth leading cause of death worldwide with an incidence of 586,000 cases and 44,000 deaths in 2020 [1]. Clinical studies are being conducted on the relationship between thyroid carcinoma and the human microbiome [71]. Increased abundance of Clostridiaceae, Neisseria, and Streptococcus was noted in stool samples of thyroid cancer patients compared to matched healthy controls. Elevated TSH and free T3 were correlated with the bacteria Porphyromonas and Streptococcus respectively [72]. Stool samples of thyroid carcinoma patients had increased levels of Proteobacteria compared to healthy controls; however, they had reduced diversity of gut microbiota [73]. In a study conducted on papillary thyroid tumor (PTC) tissues, the intratumor microbiome was significantly different compared to the adjacent normal tissue, once again proving the importance of microbes in modulating immune responses in cancer [74]. PTC tumor samples in T3/T4 stages have shown higher bacterial diversity compared to T1/T2 stages PTC tumor samples. The presence of bacterial species, Pseudomonas, Rhodomonas, and Sphingomonas in the PTC tumor samples was highly predictive of tumor invasion status, showing the role of microbiota in determining tumor behavior and prognosis [75]. Another study has revealed the importance of gut microbiome composition in determining radioiodine treatment effectiveness in PTC, but further studies are needed in this direction to validate this [76].

Gastrointestinal Cancers

The human gut is colonized by trillions of microbes, which include bacteria, viruses, fungi, and protists. Esophageal cancer is associated with Enterobacteriaceae. Gastric cancer is associated with H. pylori and other non-H. pylori organisms including Porphyromonas, Neisseria, Prevotella, Streptococcus, and Klebsiella. Colorectal cancer (CRC) is associated with increased sulfate-reducing bacteria and intestinal microbial fermentation. CRC is also associated with Fusobacterium. Liver cancer is associated with H. pylori, E. coli, and other bacteria. Pancreatic cancer is associated with H. pylori, Pseudomonas, and Fusobacterium species. H. pylori and S. typhi are associated with biliary cancer. Gastric adenocarcinoma is one of the best examples of cancers due to bacteria, especially H. pylori. Infection with H. pylori leads to gastritis, gastric ulcer, atrophy, and finally gastric cancer. The pathogenesis of most of these cancers is due to chronic inflammation; however, H. pylori can directly alter the cellular signaling pathways that affect the proliferation of gastric mucosal cells [77]. Due to this property, H. pylori is classified as a class I carcinogen by WHO [24]. H. pylori also causes MALT lymphoma.

These links are not only with single pathogens, there are several studies noting that gut dysbiosis is also associated with cancers. Gastrointestinal and non-gastrointestinal cancers are associated with gut dysbiosis secondary to repeated courses of antibiotics [78]. Dysbiosis causes cancers by inducing a chronic inflammatory state, breakdown of the normal mucosal barrier, production of secondary bile acids, or causing DNA damage. Certain bacterial species cause increased production of proinflammatory toxins, and reactive oxygen species, change signaling pathways, and can prevent antitumor immunity [79,80]. Gut microbiota is also being studied extensively for its role in cancer therapy, and antitumor immunity. The microbiome also has a role in determining the response to immune checkpoint inhibitor therapy [81] and chemotherapy [82,83]. The specific gut microbiome protects from immunotherapy-induced colitis [84,85]. Changes in the composition of gut microbiota due to radiation and chemotherapy are implicated in increased rates of radiation-induced colitis [86] and chemotherapy-induced toxicity [87]. Modulating gut microbiota, fecal microbiota therapy and probiotic organisms are being studied extensively as cancer therapies in many clinical trials [9]. Better response to PD-L-1 immunotherapy in epithelial tumors was noted with increased microbial abundance and diversity in the gut microbiome [82]. A gut microbiome with higher alpha diversity and bacteria from the Ruminococcaceae family provided specific antitumor immunity in programmed cell death protein-1 immunotherapy responders. Fecal microbial transplant to germ-free mice from the responders also led to a good response to immunotherapy [88]. Transplantation of intestinal microbiota alleviates radiation-induced toxicity as per some studies in mice [89]. Pelvic radiotherapy has demonstrated dysbiosis in involved segments of the gut, and post-radiation analysis using bacterial-epithelial co-cultures has shown increased expression of colonic tumor necrosis factor (TNF-α), IL-1β, and IL-6 in irradiated mice compared to naïve mice [90]. Administration of IL-1 beta receptor antagonist to irradiated mice has been shown to decrease dysbiosis-induced radiation damage.

Esophageal Cancer

Esophageal carcinoma is the seventh most common cancer worldwide, causing 544,000 deaths worldwide. It is responsible for one in every 18 deaths due to cancer in 2020, making it the sixth-highest in cancer-related mortality [1]. The microbiome in the oral cavity and gastrointestinal tract plays an important role in esophageal cancer etiology. Fusobacterium nucleatum in the oral cavity through the activation of chemokines is an important factor in esophageal carcinogenesis and prognosis [91,92]. A shift in the equilibrium from gram-positive to lipopolysaccharide-producing gram-negative organisms was found in reflux esophagitis and Barrett’s esophagus [93]. Campylobacter increased the expression of IL-18 in colonized tissues, leading to the progression of esophageal adenocarcinoma [94,95]. E. coli colonization is associated with the upregulation of toll-like receptors in esophageal adenocarcinoma rat models [96]. Altered microbiota in the saliva is linked to risk for esophageal squamous carcinoma. Porphyromonas gingivalis seen only in esophageal squamous cell carcinoma patients was associated with increased severity of the disease and poor outcomes [97].

Gastric Cancer

There were one million new cases of gastric cancer in 2020 with an estimated 769,000 deaths, making it the fourth leading cause of death worldwide [1]. The risk factors for gastric cancer include infection with H. pylori, dietary changes, and social habits, but changes in the microbiome are also an important factor in causing gastric cancer [98,99]. Infection with H. pylori is associated with changes in organisms residing in the stomach, leading to an increased risk for gastric cancer [100]. In a study done in China, it was noted that the incidence of gastric carcinoma is not decreased in patients who received H. pylori eradication therapy, proving the contribution of non-H. pylori factors in gastric cancer [101]. Fusobacterium, Bacteroidetes, and Patescibacteria were found in signet ring carcinoma, and Proteobacteria and Acidobacteria were noted in adenocarcinoma as per one study [102]. Numerous bacteria of the oral microbiome including Peptostreptococcus, Slackia, and Dialister were seen in gastric cancer tissue samples [103]. Gastric cancer patients also showed increased counts of Lactobacillus compared to those with intestinal metaplasia and gastritis [104]. Probiotic supplementation is found to be effective in eliminating H. pylori infection and reducing inflammation in gastric cancer patients [105,106]. Stomach bacteria also have anticancer properties with cytotoxic activity against stomach cancer cell lines [107]. The property of disrupting tumor cell membrane by H. pylori ribosomal protein-A1 can be used for cancer drug delivery [108].

Colorectal Cancer

Colorectal is the third most diagnosed cancer and the second leading cause of death with an estimated 935,000 deaths and 1.3 million new cases in 2020 [1]. There is emerging evidence of the changes in the composition of microbiota in colorectal cancers compared to normal healthy mucosa [109,110]. In these studies, it was shown that there was an overrepresentation of certain bacteria including Lactococcus and Fusobacterium while Pseudomonas and Escherichia were underrepresented [111]. Transplantation of stool samples from patients with colorectal cancer to healthy mice led to an increased number of polyps and intestinal dysplasia compared to the mice that received stool samples from healthy individuals [112]. Certain bacteria like Fusobacterium nucleatum were detected in frozen specimens from patients with colon cancer (Table 2). These bacteria were not only detected in primary tumors but also in liver metastases from colon cancer [113,114]. Gut microbiome-derived extracellular vesicles (EVs) were significantly different in colorectal cancer patients compared to control subjects. These microbiota-derived EVs can be used for predicting cancer prognosis and for treatment [115,116]. Treatment with conventional drugs including 5-fluorouracil, irinotecan, and oxaliplatin in mice led to changes in gut microbiota. Germ-free mice tolerated chemotherapy better and had fewer side effects. Inhibiting certain enzymes also prevents intestinal toxicity from chemotherapy. These effects are also observed with other treatments including radiotherapy and immunotherapy [117]. Identifying the pattern of dysbiosis can aid in designing microbiome biomarker screening tests [118,119].

Primary Liver Cancer

Primary liver carcinoma was the third leading cause of cancer-related deaths worldwide in 2020, with approximately 906,000 new cases and 830,000 deaths [1]. The liver is situated near the gut microbiome and the gut-liver axis plays a prominent role in liver carcinogenesis. Gut dysbiosis has been associated with hepatocellular carcinoma (HCC) [120]. Bacterial metabolites from the gut can reach the liver through the portal venous system, leading to hepatotoxicity. Gut microbes can change primary bile acids to secondary bile acids, leading to DNA damage, depletion in tumor-suppressing micro-DNA, alteration in numbers of NK T cells, and carcinogenesis [121-123]. Obesity resulting in nonalcoholic steatohepatitis (NASH) is also indirectly associated with gut dysbiosis leading to HCC [124].

Gallbladder Cancer

Gallbladder cancer is rare and has a poor prognosis [125]. Salmonella species were directly associated with gallbladder cancer, especially in countries with a high incidence of these organisms [17,18]. Chronic typhoid carriers are more likely to die of hepatobiliary cancers [126,127]. Certain bacteria like Helicobacter are associated with this cancer in recent years [113]. In studies, metagenome analysis from the blood samples of biliary cancer patients showed an altered microbiome compared to control groups. Further studies with matching controls are needed to further confirm these results [128]. Studies have also shown the role of bile microbial dysbiosis in gall bladder cancers [129].

Pancreatic Cancers

Pancreatic cancer is the seventh leading cause of cancer-related deaths worldwide and the third leading cause of death in the United States [130]. It has a very poor prognosis and deaths from it are equal to the number of cases due to a lack of screening techniques leading to delayed presentation [1]. The pancreas was once thought to be a sterile organ; however, recent gene sequencing techniques have identified bacteria not only in normal pancreatic tissues but also in pancreatic ductal adenocarcinoma, showing the potential role of the microbiome in homeostasis [131,132]. Lipopolysaccharide (LPS) from oral microorganism Porphyromonas gingivalis triggers an innate immune response and leads to inflammation by the release of proinflammatory cytokines. The immune response involves the recognition of toll-like receptor 4 (TLR 4). These TLRs are highly expressed in human pancreatic cancer leading to inflammation [133,134]. Oral microorganisms including Porphyromonas and Fusobacterium are found to be linked to pancreatic cancers [135,136]. It was shown that bacteria from the gut migrate to the pancreas. Ablation of gut bacteria with antibiotics in mice was protective against pancreatic ductal carcinoma progression and it also upregulated PD-1 expression [132]. In one study, it was shown that the presence of certain bacteria in pancreatic ductal adenocarcinoma led to resistance to a chemotherapeutic drug, Gemcitabine, proving the role of the microbiome in the treatment of cancers [137]. In addition to intratumor and oral dysbiosis, gut dysbiosis also plays a prominent role in pancreatic cancers. The composition of gut microbiota is much different in pancreatic ductal adenocarcinoma patients compared to healthy controls [138].

Breast Cancer

Breast cancer remains the most diagnosed cancer in women worldwide with 2.3 million cases leading to 685,000 deaths [1]. The microbiome has been identified as one of the risk factors for breast cancer in recent studies. Fresh frozen breast tissue samples showed distinct microbiome composition compared to controls [139,140]. Fusobacterium species in breast cancer increased cell proliferation and protected tumors from the immune system. Gut dysbiosis is associated with breast cancer by its effect on estrogen metabolism [141-143]. Further studies are needed to prove this association. Oral dysbiosis in the form of periodontal disease has also been shown to be associated with breast cancer [144]. Nucleic acid signatures of many viruses including polyoma, papilloma, and herpes viruses were noted in different breast cancer subtypes. Several specific fungi and parasitic signatures were also noted in these samples [145].

Genitourinary System Cancers

Genital Cancers

Cervical cancer is still a leading cause of death worldwide, especially in low-income group countries [1]. With widespread vaccination and routine screening, the incidence and mortality have decreased in developed nations. Cervical cancer is caused by HPV infection, and changes in the cervical microbiome were also noted in women with cervical cancer [146,147]. Studies reveal the association of changes in the vaginal microbiome with persistent HPV infection leading to high-grade dysplasia and cervical cancer. Endometrial cancer is a common gynecological cancer that is usually diagnosed in post-menopausal women. It is divided into two types based on the relationship with estrogen. The endometrial microbiota is responsible for maintaining homeostasis and proper functioning of the uterine environment. Differences in the composition of endometrial microbiota in endometrial cancer patients compared to controls were noted in several studies [148]. Studies proved the role of lipopolysaccharides, secondary bile acids, and short-chain fatty acids in ovarian cancer [149].

Prostate Cancer

Prostate cancer is affected by both estrogen and androgen levels. The Global Cancer Observatory (GLOBOCAN) estimated about 375,000 deaths worldwide with 1.4 million cases in 2020 due to prostate cancer [1]. Inflammation is a risk factor for the development of prostate cancer. Prostatic fluid is antimicrobial, and no specific prostate microbiota have been identified so far. There have been recent studies showing the importance of the urinary microbiome in prostate inflammation and cancer [150]. They reported changes in the microbiome of urine and seminal fluid in patients with chronic prostatitis, which is a risk factor for cancer. Changes in the microbiome in the gastrointestinal tract and oral cavity were also noted in prostate cancer patients [151-153].

Bladder Cancer

Bladder cancer is the 10th most common cause of cancer and led to 213,000 deaths worldwide as per GLOBOCAN 2020 statistics [1]. The risk factors for bladder cancer include tobacco smoking and exposure to harmful chemicals. It is common in areas with a high prevalence of Schistosoma haematobium [154]. The urinary microbiome is considerably different in various disease states including diabetes mellitus, neuropathic bladder, urinary incontinence, and others [155-157]. A microbiome study conducted on urine samples of urothelial carcinoma patients showed increased Streptococcus, Pseudomonas, and Anaerococcus compared to controls [158]. Another study conducted on the urinary microbiome in urothelial carcinoma patients did not show a significant difference in microbiota composition but identified an overrepresentation of several operational taxonomic units (OTUs) [159]. Further studies are needed to exactly characterize the urinary microbiome in bladder cancer patients.

Skin Cancers

According to GLOBOCAN statistics, about one million new non-melanoma skin cancer cases were recorded in the year 2020, leading to 64,000 deaths [1]. Microbial dysbiosis was noted in skin swabs of squamous cell carcinoma patients who were on immunosuppressants after an organ transplant [160]. Staphylococcus aureus and Cutibacterium were associated with squamous cell carcinomas of the skin [161,162]. Some studies have also found an association between HPV and non-melanoma skin cancers [163]. In vitro studies have revealed that changes in gut and skin microbiome were associated with the progression of melanomas [164].

Overall, dysbiosis with increased bacteria is associated with various cancers including oral, esophageal, breast, lung, liver, intestinal, and others (Table 1). The mechanisms by which specific bacteria cause cancers are well studied in the gastrointestinal microbiome, especially the colon microbiome (Table 2).

**Table 1:** Examples of specific organisms associated with dysbiosis and cancers

Type of cancer	Cancer microbiome
Esophagus	Campylobacter spp. [96,97], Escherichia coli [98]
Breast	Streptococcus, Propionibacterium, Escherichia coli, Staphylococcus epidermidis [142,143]
Oral	Porphyromonas gingivalis, Fusobacterium nucleatum [165,166]
Lung	Granulicatella, Streptococcus, Abiotrophia [167]
Liver	Enterococcus, Ruminococcus, Bacteroides, Phascolarctobacterium, and Oscillospira [168]
Colon	Fusobacterium nucleatum, Escherichia coli, Bacteroides fragilis, Porphyromonas [169-172]
Gall bladder	Fusobacterium nucleatum, Escherichia coli, and Enetrobacter spp. [173]
Prostate	Anaerococcus lactolyticus, Varibaculum cambriense, and Propionimicrobium lymphophilium [174]
Pancreas	Proteobacteria, Bacteroidetes, and Firmicutes [175-177]

**Table 2:** Mechanisms by which some bacteria cause colorectal cancers

Organism	Mechanism of action
Enterococcus fecalis	DNA damage from increased production of hydroxyl radicals [178]
Peptostreptococcus anaerobius	Increases reactive oxygen species and cell proliferation [179]
Salmonella	Activation of STAT3 and the Wnt signaling pathway [180]
Fusobacterium nucleatum	Forms protein complex with beta-catenin and modulates its expression affecting the Wnt pathway [181]
Campylobacter species	Produces genotoxins leading to DNA damage [182]
Escherichia coli	Produces colibactin leading to DNA double-strand breaks and activation of DNA damage checkpoint pathway [183]
Bacteroides fragilis	Produces Bacteroides fragilis toxin [184]
Helicobacter pylori	Due to cytotoxin-associated gene, reactive oxygen species [185,186]
Clostridium septicum	A hypoxic and acidic tumor environment favors its growth and plays a role indirectly [187]

## Conclusions

Cancer affects at least one person in every family and the effect it leaves on any family is significant. In 2020, 19.3 million new cases of cancer were detected as per GLOBOCAN statistics, and the incidence has been increasing over the past few decades. GLOBOCAN estimates that 28.4 million new cases of cancer will be diagnosed by 2040 worldwide. Cancers can be cured if diagnosed and treated early. However, the problem is that we lack effective screening strategies to diagnose cancers at an initial asymptomatic stage and most cancers are typically diagnosed at an advanced stage, leading to increased mortality. The association of the microbiome with different cancers can help us devise better screening strategies and develop new treatments. Targeting our genome with chemotherapy or immunotherapy is associated with significant side effects leading to early cessation of cancer treatment. The human genome is 99.9% identical within the species, but we are almost 80-90% different from each other in terms of the gut and other microbiomes. This presents a wonderful opportunity for targeting the microbiome instead of our genome for designing new screening techniques and cancer drugs.

Changes in microbiota composition with probiotics, antibiotics, and other treatments can be used to develop specific microbial signatures. Studies showing specific microbial signatures in cancers may help us develop specific markers correlating disease severity and prognosis. We can use these signatures to treat cancers with specific organisms that are proved to have anti-cancer or anti-inflammatory properties. With the current advances in technology, we can identify microbiota changes associated with different cancers, but significantly more research is needed to develop microbial biomarkers, which can further revolutionize the prevention and help us with early diagnosis of the cancers. Studies in mice have shown that fecal microbial transplantation with specific bacteria reduces radiation-induced damage, so the identification of these microbes can help identify the radiosensitivity of the patient’s cancer. We can use these microbes in the treatment for reducing damage in dysbiosis patients. It was also noted that post-radiation mice had specific microbial signatures, and this can help us identify microbes hypersensitive to radiotherapy and prevent radiation-induced side effects such as proctitis.

Immunotherapy has improved the prognosis of many advanced cancers and changed the perspective of cancer treatment in many ways. Studies have proven that the effectiveness of PD-L-1 immunotherapy decreases in patients treated with antibiotics, and oral probiotics have shown improved response to immunotherapy. We can use this property and make the treatment more effective by supplementing good bacteria. Gut microbiota can also modulate the efficacy and toxicity of chemotherapy. Even though studies show the association of microbiome with cancer development and susceptibility to treatments, the complex mechanisms are still unknown and in-depth research is further needed. Fungi and viral microbiome are yet to be explored. Finally, microbiome patterns may be used as a marker for cancer diagnosis, a prognostic marker for cancer survival, and a predictive marker for treatment response. Microbes can probably be used for preventing cancer, increasing the effectiveness of treatment in already diagnosed cancer, or as an add-on treatment, especially as probiotics. Modified EVs from the microbiome can have applications in cancer drug delivery in the future, which can precisely focus on the organ or tissue, thereby providing high-quality organ-specific treatment leading to lesser side effects. In conclusion, a better understanding of the cancer microbiome and its response to different treatments can provide great opportunities for curing the cancer pandemic.
